# Experience of midwives in providing care to labouring women in varied healthcare settings: A qualitative study

**DOI:** 10.4102/hsag.v26i0.1524

**Published:** 2021-05-31

**Authors:** Marie Hastings-Tolsma, Annie Temane, Oslinah B. Tagutanazvo, Sanele Lukhele, Anna G. Nolte

**Affiliations:** 1 Louise Herrington School of Nursing, Faculty of Nursing/Midwifery, Baylor University, Dallas, Texas, United States of America; 2 Department of Nursing, Faculty of Health Sciences, University of Johannesburg, Johannesburg, South Africa; 3 Department of Midwifery Science, Faculty of Health Sciences, University of Eswatini, Mbabane, Eswatini; 4 Netcare Education, Netcare, Johannesburg, South Africa

**Keywords:** qualitative research, midwifery, childbirth, labour, sub-Saharan Africa

## Abstract

**Background:**

Midwives are essential to timely, effective, family-centred care. In South Africa, patients have often expressed dissatisfaction with the quality of midwifery care. Negative interpersonal relationships with caregivers, lack of information, neglect and abandonment were consistent complaints. Less is known about how midwives experience providing care.

**Aim:**

This research explored and described the experiences of midwives in providing care to labouring women in varied healthcare settings.

**Setting:**

Midwives practicing in the Gauteng province, South Africa, in one of three settings: private hospitals, public hospitals or independent maternity hospital.

**Methods:**

A convenience sample of midwives (*N* = 10) were interviewed. An exploratory and descriptive design, with individual semi-structured interviews conducted, asked a primary question: ‘How is it for you to be a midwife in South Africa?’ Transcribed interviews were analysed using thematic coding.

**Results:**

Five themes were found: proud to be a midwife, regulations and independent function, resource availability, work burden and image of the midwife.

**Conclusion:**

Midwives struggle within systems that fail to allow independent functioning, disallowing a voice in making decisions and creating change. Regardless of practice setting, midwives expressed frustration with policies that prevented utilisation consistent with scope of practice, as well as an inability to practice the midwifery model of care. Those in public settings expressed concern with restricted resource appropriation. Similarly, there is clear need to upscale midwifery education and to establish care competencies to be met in providing clinical services.

**Contribution:**

This research provides evidence of the midwifery experience with implications for needed health policy change.

## Introduction

Midwives are an essential part of healthcare who offer timely, effective and family-centred services. The care provided by midwives has a major effect on the well-being of mothers and babies (Halldorsdottir & Karlsdottir 2011:806). This is particularly true in many low- and middle-income countries where midwives provide the preponderance of maternity care (UNFPA 2011). Despite the potential powerful influence of midwives, many challenges are being faced whilst providing quality care including the need to reduce maternal mortality rates, growing litigation and a shortage of resources.

In South Africa, an institutional maternal mortality rate of 134 per 100 000 live births – a rate almost exclusively from public healthcare facilities (Department of Health Republic of South Africa 2017), far exceeds the international goal for fewer than 38 deaths per 100 000 (UN 2015). Furthermore, 60% of these deaths have been deemed preventable with avoidable factors and substandard care as major contributors. It has been estimated that two-thirds of maternal and newborn deaths, as well as stillbirths, could be eliminated by 2035 if midwifery healthcare standards were improved (Nove et al. 2021:e24). Additional contributing factors include failure to accurately assess patients, delays in referral, failure to follow standard protocols and poor monitoring (Moodley et al. 2014:58). The problem is further complicated by excessive surgical birth rates – particularly in the private sector where rates have exceeded 60% for more than a decade (Council for Medical Schemes 2017/2018). Failure to promote physiologic birth lies squarely on the World Health Organization (WHO), which currently does not recommend an ideal Cesarean birth rate (WHO 2015a), as well as on midwives and physicians who have failed to combat unnecessary birth interventions.

Midwives in South Africa have been involved in an increasing number of medico-legal cases reflecting concerns about the nature and quality of care. At the current time, the Gauteng province – the most populous province in South Africa, faces medico-legal claims amounting to R29 billion with the vast majority for alleged medical negligence related to infants afflicted with cerebral palsy because of poor intrapartum management in public facilities (Bloom 2019). Such claims have depleted monies needed to ensure adequate staffing, equipment and facilities.

Whilst increased attention is being directed to address the root causes for medico-legal action, little attention has been directed at examining midwifery-related factors. Surprisingly, recent examination of rising medico-legal maternity related cases failed to make any mention of midwives (Oosthuizen & Carstens 2015; Taylor et al. 2018).

Over the last few years there has been a sharp deterioration in healthcare at public hospitals and clinics in the Gauteng province, marked by shortages of medicines, collapsing infrastructure, broken equipment, inadequate provision of staff and misuse and misallocation of funds (Maphumulo & Bhengu 2019:2–3). A lack of adequate facilities and resources leaves midwives feeling drained and exhausted, struggling to cope with an overwhelming workload.

Many hospitals in South Africa that provide services to thousands of pregnant women annually, experience a shortage of qualified midwives. To combat shortages in the public sector, administrators often deploy nurses who are not midwives to maternity sections. Ongoing midwifery shortages and site reassignments result in midwives who are fatigued, burnt out, performing at less than full capacity and unable to promote improved perinatal outcomes (Matlala & Lumadi 2019:7). These problems are compounded by a physician shortage with many of those who are available opting for service in private facilities leaving the public system – which serves the vast majority of the population, chronically short staffed. Because of the physician shortage, midwives play a crucial role in reducing maternal mortality and morbidity.

Research from South Africa has found patients with expressed dissatisfaction regarding the quality of midwifery care. More than half of women who have given birth in the public sector reported ‘narratives of distress’ (Chadwick, Cooper & Harries 2014:862). These narratives included consistent complaints of negative interpersonal relationships with caregivers, lack of information, neglect and abandonment. These same concerns have been voiced by women in other research, along with concerns about denial of a labour companion, being left alone and denial of care (Hastings-Tolsma, Nolte & Temane 2018:e47–e48; Jewkes, Abrahams & Mvo 1998:1785–1791; Jikijela, James & Sonti 2018:8; Kruger & Schoombee 2010:87; Maputle & Nolte 2008:60–61; Sengane 2013:6–8). Alternately, midwives interviewed regarding experiences in managing women in labour, viewed their care as midwifery or institution-centred rather than woman-centred (Bradley et al. 2016:157; Lambert et al. 2018:256; Maputle & Hiss 2010:12). Midwives were observed to have told mothers what to do and how to behave, imposing their authority and responsibility to ensure that rules were followed.

The dissatisfaction with midwifery care processes has been documented for well over two decades (Jewkes et al. 1998:1794) and is consistent with global report of a lack of care in nursing (Scott 2014:177). In South Africa, midwives wear maroon epaulettes with a green bar to signify the specialty although in earlier times, midwives wore a green epaulet. The devices symbolise that the midwife is professionally qualified and can be trusted to provide top quality care. Prior to 1856, nurse training comprised of a 4-year diploma at a hospital-based nursing college, which was viewed as both rigorous and woman-centred. This training was subsequently replaced by a comprehensive 4-year qualification (including general nursing, midwifery, community and psychiatric nursing), which can be completed through a nursing college diploma or a university degree (Blaauw, Ditlopo & Rispel 2014). The current comprehensive training of nurse midwives in South Africa mandates an emphasis on four specialties within nursing. This was carried out to meet demands of the public health sector. This requirement has contributed to beliefs that new midwives are neither skilled nor are they competent in midwifery, thus they lack practical training (Webbelink 2019:103–105).

Working primarily in public, private and independent settings, midwives often face varied challenges including overcrowded labour wards, a lack of resources, misutilisation and a shortage of health personnel. These challenges correspond with growing reports by labouring women of substandard midwifery care in South Africa, particularly in public hospital settings (Human Rights Watch 2011), and escalating global recognition of neglectful, abusive and disrespectful treatment of women during childbirth, particularly in low income, low resource countries (Bohren et al. 2015). These factors likely influence the ability of midwives – regardless of employment setting, to engage in the care of women who foster improved perinatal outcomes and satisfaction. There is a dearth of research that explores the experience of midwives in caring for labouring women across practice settings and further study is required.

### Aim

Midwives’ relationship with the childbearing woman is a major source of job motivation and satisfaction (Curtis, Ball & Kirkham 2006:29). This relationship is the very essence of midwifery care and defines its distinctive nature (Leinweber & Rowe 2010:82–83), although little is known about how midwives experience midwifery practice under varied circumstances. The aim of this research was to explore and describe the experiences of midwives in providing care to labouring women in varied healthcare settings.

## Study design

This qualitative, exploratory, descriptive and contextual study sought to gain a better understanding of midwives’ experiences of caring for women during birth. A constructivist approach draws on the belief that there is no single reality; rather efforts are made to elicit participant views of reality (Bergman et al. 2012:1). This approach allowed researchers to conduct an in-depth examination of how midwives make meaning out of their experiences. The consolidated criteria for reporting qualitative research (COREQ) guidelines were used to guide report of findings (Tong, Sainsbury & Craig 2007).

### Participants

A purposive, convenience sample of midwives (*N* = 10) who cared for women during childbirth in the Gauteng province in South Africa were recruited using snowball or chain-referral sampling technique. Recruitment was through personal contact with investigators. The inclusion criteria included ability to understand and speak English, currently engaged in midwifery practice in South Africa and attending births in either the public, private or independent maternity hospital setting. These settings were selected as they are the primary sites where South African midwives are employed although fewer are known to work in an independent fashion. Exclusion criteria included student midwifery standing or retired. Recruitment continued until saturation was achieved.

### Data collection and setting

One of the investigators (M.H.T.), with no prior participant relationship, conducted all face-to-face interviews. The semi-structured, in-depth interviews were conducted when the midwife was not on duty and at a time of participant convenience. Interviews lasted from 35 min to 70 min and were conducted in a quiet room where there were no external disturbances. One central question was asked, ‘How is it for you to be a midwife in South Africa?’ When appropriate, additional questions were asked to obtain a deeper understanding of the midwives’ experiences (Table 1).

**TABLE 1:** Representative prompts from the interview guide.

Overarching question	How is it for you to be a midwife in South Africa?
Additional prompts	How did you choose to work with labouring women?
	How do you feel in providing care to labouring women?
	If you could make changes in how care is provided to labouring women on (in) your unit (practice), what would those changes be?
	What makes you most proud in providing care to labouring women?
	If you or someone you love were going to have a baby, where would you think it best to give birth?

Interviews were recorded using a Sony ICD-UX560 digital audio-recorder with field notes maintained. Two midwives participated in a pilot study to determine whether the questions were clear. These two participants were subsequently included in the study as there were no concerns voiced. Participants were interviewed until no new information emerged.

### Data analysis

The data analytic process detailed by Braun and Clarke (2006) was used to identify, analyse and report patterns or themes within the data. Researchers familiarised themselves with the data, generated initial codes, searched for themes, reviewed themes and then defined and named the themes with the final production of a report.

Specifically, audiotaped interviews were transcribed verbatim. Because descriptions of midwifery care during birth often contained both English and African and Afrikaans languages, transcripts were reviewed for meaning by the researchers who were native to South Africa (A.N., A.T.). Data were subsequently analysed by the same two researchers (M.H.T., A.N.) using thematic analysis (Vaismoradi, Turunen & Bondas 2013). Researchers read and re-read all participant transcripts to better understand meaning and to identify themes. This iterative process involved an initial thematic analysis achieved through manual coding consistent with the aim of the study, identification of codes grouped into themes to foster interpretation and the identification of representative quotes to illustrate themes. Theme categories and subcategories were identified by the two researchers, after which consensus discussions were held about the findings.

Two of the researchers engaged in data analysis were midwives (A.N., M.H.T.). One of these researchers (A.N.) was a registered midwife in South Africa with extensive clinical and academic midwifery experience; the other (M.H.T.) was a research collaborator living in South Africa and who had significant academic and clinical midwifery experience. Consistent with the approach, transcript analysis focussed on context, integrating manifest and latent content without use of a linear process and excluded member checking. Where there were differences amongst researchers in interpretation of findings, prolonged engagement was utilised until agreement was reached.

#### Trustworthiness

The criteria for trustworthiness in qualitative research as described by Lincoln and Guba (1985) were followed, namely credibility, transferability, dependability and confirmability. Purposive sampling was used and ensured engagement of participants capable of providing detailed information in response to the research question using 1:1 in-depth interview and field notes to ensure credibility and transferability. Dependability was achieved by the code-recode method of analysis, where data were coded over an extended time period to ensure consistency in coding. Confirmability was ensured by documenting direct quotations, as well as a confirmability audit.

### Ethical considerations

The four principles of the Medical Research Council of biomedical ethics were followed (South African Medical Research Council 2018). Written informed consent was obtained prior to study participation. Ethical clearance was obtained from the University of Johannesburg’s Health Science Ethical Committee (AEC51-01-2012), as well as the University of Colorado Multiple Institutional Review Board (#12-1458).

## Results

### Demographics

Participants consisted of 10 practicing midwives between the ages of 27 and 54 years with varied race or ethnicity: five were black, three mixed race and two white people. All were female, married and had achieved post-baccalaureate level education or higher. All were employed in either the public (*n* = 6), private (*n* = 3) or independent maternity (*n* = 1) hospital setting.

### Themes

Five main themes were identified and included proud to be a midwife, regulations and independent function, resource availability, work burden and image of the midwife (Table 2).

**TABLE 2:** Themes and categories: Experiences of midwives (*N* = 10).

Themes	Categories
Proud to be a midwife	Self-esteem Diversity of practice Interesting Care of healthy people Professionalism
Regulations and independent function	Relationship with physicians Authorities
Resource availability	Shortage of physicians Shortage of midwives Environmental equipment/supplies Administrative support Space for family-centred care
Work burden	Midwife: Patient ratio Influx of immigrants Communication challenges
Image of midwife	Respectful care Patient trust Public perception

#### Proud to be a midwife

Participants expressed pride in being a midwife stating that it boosted *self-esteem*, provided *diversity* and was *interesting* as they cared for *healthy people*. When demonstrating competence, respect followed with *professionalism* bolstered:

‘I am very proud of where I work because [*of*] the influx of patients and the reasons why they come. I know that we are doing something right. I would advise anyone to [*birth*] with us.’ (Participant 4, female, public)

‘Midwifery is interesting … there is no routine … something different all the time. It gives you joy and you feel good seeing a positive outcome at the end of pregnancy.’ (Participant 9, female, public)

‘Midwives get a lot of respect in my institution. I think it also depends on you, as a professional, how you carry yourself, how competent you are and you know you are empowered and up-to-date … if you are empowered you get a lot of respect …’ (Participant 7, female, private)

#### Regulations and independent function

The *relationship with physicians* and regulations by *authorities* both contributed to this theme. Midwives in public hospitals expressed a greater sense of independence in decision-making whilst midwives in private hospitals voiced a more restricted scope of practice. Those in private maternity hospital settings were able to engage in independent management and shared decision-making with women:

‘In private hospital, patients belong to the obstetrician. Midwives hold the birth for the obstetrician. It’s a lot of frustration … we have to depend on the decision of [*physicians*].’ (Participant 2, female, private)

‘Midwives can do everything themselves even if there are complications, they can do it without a [*physician*].’ (Participant 10, female, public)

‘I feel more independent … making decisions that I feel are good for my clients.’ (Participant 8, female, independent maternity)

Despite feeling independent, participants working in the public hospital setting felt that hospital authorities would not allow midwives to make decisions and create needed change:

‘I don’t think administrators give us authority to make changes. We get regulations from above … this is how we [*midwives*] are going to function … these are the protocols that you follow.’ (Participant 5, female, public)

Finally, there were concerns about the management of medical aid plans where private facilities were viewed as receiving disproportionately greater compensation despite seeing fewer patients. Physicians’ attitudes towards birth was observed to be a part of the problem as well:

‘There is a huge dichotomy between private and public hospitals. Private hospitals take 80% of the monies but they don’t do midwifery management because it doesn’t actually pay. It is not surgery or high care medicine. But private hospitals are overloaded as well, which is why the Caesar rates are so high in both private and public settings.’ (Participant 4, female, public)

‘One [*physician*] told me that “the vagina is made for sex and not for delivery.”’ (Participant 10, female, public)

#### Resource availability

Midwives, especially in public hospitals, often had to function without the necessary resources with a *shortage of physicians and midwives*, inadequate *equipment and supplies*, inadequate *administrative support* and lack of *space for family-centred care*.

The shortage of physicians differed from hospital to hospital with variable availability. Generally, physician availability was better in the private setting with uneven access in public facilities:

‘We do have [*physicians*] around for a certain period and time, and they are accessible if you really need them.’ (Participant 6, female, public)

‘If there are complications, it’s difficult to get hold of the [*physician*].’ (Participant 4, female, public)

Participants believed there was a significant shortage of midwives resulting in increased litigation. They were also of the opinion that the shortage was worse in public hospitals where high numbers of births exacerbated the problem. The shortage of midwives and heavy workload contributed to difficulty in providing individualised care and resulted in women often being left alone for long periods of time:

‘[*B*]ecause of workload and staff shortages you cannot give [*women*] your entire support and care. In my hospital, there are two professional midwives working day shift and we are very busy – sometimes more than 20 births.’ (Participant 3, female, public)

‘In private hospitals you may be looking after two patients at the same time … there is less work – so you are very close to the patient. But in public hospitals you are not close to any patient. I often have 7 patients. I may be busy with this one and the other one is pushing … there just isn’t time … you literally run from bed to bed catching babies.’ (Participant 6, female, public)

Heavy midwifery workloads contributed to feelings of exhaustion:

‘Sometimes you feel that you work without a break, without lunch, without tea … and by the time you go home, you can’t even eat or wash … that is how exhausted you are.’ (Participant 9, female, public)

The lack of physical resources including equipment and supplies was a further burden for effective midwifery function. Midwives who worked in public hospitals complained about their lack of equipment and supplies often exacerbated by unexpected immigrants who made it difficult to plan for enough supplies:

‘We don’t have resources. Some patients bring their own bedding and sheets from home, in addition to their own pads … and we appreciate that. We start off with many supplies but run out during the fiscal year and have to do without. Sometimes we don’t even have heated water.’ (Participant 9, female, public)

‘We start with enough supplies, but patients coming across the border to give birth makes it hard to plan and we run out. We don’t have enough beds and even if they sit in a chair or whatever … they just give birth where they are ….’ (Participant 1, female, public)

The lack of resources made care, as well as patient education, difficult and was an embarrassment to midwives:

‘We don’t have descent bedding to give to our patients … every time you have to explain yourself.’ (Participant 9, female, public)

‘We used to have some leaflets on some danger signs and what to prepare for when women go to hospital but we don’t have that anymore. Sometimes we run out of paper. I just took out my own money to go buy paper so that we can make copies ….’ (Participant 4, female, public)

Midwives were frustrated that hospital management did not support them when they had to work under difficult circumstances. Similarly, management was viewed as doing little to help improve and empower midwives and they viewed management to be judgmental:

‘The way management treats the staff … instead of coming to support you, they are pressing you. When something goes wrong – instead of wanting to find out what went wrong they are judgmental, [*rather*] than helping you on how to make yourself better and how to improve.’ (Participant 9, female, public)

Midwives working in the public setting also noticed that generally it was not possible for a companion to accompany the labouring woman, citing lack of space, privacy concerns and security as the primary reasons. Conversely, midwives in the private and private maternity hospital settings noticed family inclusion, as desired:

‘There is no space … labor beds are close together with several patients sharing one large open labor ward … so there can be no privacy. We do not allow family in because it’s an open setting and we look at the dignity of the patient … we protect the other patients who have a low threshold for pain because they undress and cry and vomit and so on.’ (Participant 3, female, public)

Culture was observed to be an important factor in providing family-centred care during labour and birth:

‘Some [*women*] don’t want anyone with them … they prefer to be alone. Also, some African men are scared of birth … they don’t want to be present. Culture plays a role.’ (Participant 9, female, public)

#### Work burden

Participants observed three factors that contributed to feelings of *work burden* with low wages, a high *midwife to patient ratio* and *an influx of immigrants* giving birth in public facilities, which often presented *communication challenges*. Large numbers of patients – particularly immigrants, created a sense of being overwhelmed, compounded by wages that were poorer than in the private sector. Language barriers further exacerbated participant frustration in providing care. Burdens in the public setting contributed to midwives’ desire to work in the private sector despite a restricted midwifery scope:

‘Most of the time illegal immigrants do not book for antenatal care or place of delivery – but there is nothing we can do … we have to deal with it. You continuously take in patients whether the hospital is full or there is space.’ (Participant 4, female, public)

‘[*P*]eople come from wherever and [*speak*] a language you don’t understand … you try to make sign language but how can you help somebody if you cannot really communicate?’ (Participant 7, female, public)

‘… I would work in the private sector if I could … everyone knows the pay is better … money talks ….’ (Participant 6, female, public)

#### Image of the midwife

The provision of *respectful care, patient trust* and *public perception* was identified by participants as factors in the image of midwives. Participants, often accused of being disrespectful to women, were aware of this image. Some believed that this behaviour towards women was justified to ensure positive birth outcomes. Other participants felt workload was the basis for neglectful, disrespectful care, creating patient mistrust of midwives:

‘Sometimes you find during [*birth*] there’s a problem where maybe a patient don’t listen to the midwives … they don’t want to push or something like this. And sometimes you raise your voice … it’s because you are concerned about that baby. Some of the midwives call it “therapeutic smacking” … but you know after the [*birth*] you need to soften and explain to the mother why you had to raise your voice.’ (Participant 5, female, public)

‘Some [*patients*] will say they haven’t been treated well. Some will complain that midwives shout at them … that they are rough. Many in the public – particularly blacks, find midwives very abusive because they smack them.’ (Participant 2, female, private)

‘You hear about midwives raising their voices to patients …some are rude or mean. I think the workload and being tired and getting patients who don’t belong in this area (*South Africa*) has a lot to do with the midwife’s attitude … how they come across and speak to people … those with low tempers tend to react harshly. But in my institution, it’s totally not allowed.’ (Participant 3, female, public)

Midwives working in the public setting noticed that many women did not want to give birth in hospital. The system was deemed to be authoritarian with little regard for shared decision-making:

‘Many patients [*give birth*] at home because they deliver before transport arrives. Some wait because they are frightened of midwives. Once they give birth, then they call the ambulance. They basically come into the hospital with the baby because they need the wellness card and they know they need a birth certificate to access the road to health care in South Africa.’ (Participant 4, female, public)

As participants reflected on the basis for harsh treatment of women by midwives, they observed lack of passion for midwifery compounded by inadequate midwifery education. Participants were also aware of how midwifery behaviour towards women might influence student midwives in clinical training:

‘Maybe 4 out of 10 midwives don’t really have a passion for midwifery. It is a challenge when people go into midwifery because they have nothing else to do … it is just a job … they have no desire to care for people … it is not a calling to them. [*When not a calling, these midwives often*] come to harass people. I see they don’t treat women well … they just talk in a manner that shows they don’t care … they shout at patients … they hit patients. I think they do these things because they are frustrated and are not prepared to serve. This is what creates the problem for how the public see midwives.’ (Participant 1, female, public)

‘I think that midwifery must change … we need more training as well as more midwives. And the pay must change … midwives would work in a private facility if they had a choice [*because*] they pay more money.’ (Participant 7, female, private)

‘Midwifery students have a problem – they are distressed when they see midwives smacking people – abusing them. The midwives say the patients don’t listen – so they must learn.’ (Participant 5, female, public)

An additional factor contributing to the poor image of midwifery was believed to be public confusion regarding the role of midwives. This confusion extended to physicians who serve as gatekeepers to the healthcare system:

‘I think one of the biggest challenges for midwives in South Africa is that people do not know how we are different from nurses. There are a great number of people who have no idea what a midwife is, including [*physicians*] who probably think of midwives more as nurses.’ (Participant 2, female, private)

‘In the old days, [*physicians*] viewed midwives as the people that taught them. Some older physicians still talk about the “green epaulet girls” … the midwives who worked primarily in the district services. They viewed them as phenomenal … that they [*midwives*] knew what they were doing. Now they call [*midwives*] people who merely do the observations and don’t do them very well.’ (Participant 1, female, public)

Finally, participants were queried regarding where they themselves would choose to give birth if medical insurance was not a consideration. Despite beliefs that the public did not understand the role of the midwife, participants working in the public setting were reluctant to choose receiving care in public facilities expressing preference for the private hospital setting with a physician as the birth attendant. Midwives in the private hospital setting were mixed in a desire for care in private hospitals with a physician or in an independent maternity hospital with a midwife. None of the participants gave voice to preference for birth with a midwife in the public setting:

‘… I would choose a gyne for my pregnancy care and birth because the gyne will look at the baby, take me to sonar and check the well-being of the baby. Midwives in the public setting don’t have sonar and stuff … it is a matter of having the resources.’ (Participant 10, female, public)

‘The difference between public and private is all about money. If you are a woman and you are very much scared of pain – especially if you are a primigravida, you will hear people telling you that if you are in a private you can just tell the [*physician*] “I am tired” and then, the [*physician*] will give you the date to take out the baby.’ (Participant 5, female, public)

## Discussion

This research highlighted several findings of concern. Whilst policy to move birth to the institutional setting has likely been instrumental in the reduction of maternal mortality and morbidity, it has altered the psychosocial elements crucial in providing a midwifery model of care (Bradley et al. 2016:127–158). Institutional emphasis on technological birth – irrespective of setting has left midwives struggling for a voice in systems dominated by the medical model. As the global need for midwifery care continues to grow, there is a need to reconsider the extent to which midwives participate in the establishment of care practices.

The first theme, **proud to be a midwife**, reflected *self-esteem, diversity of practice, the interesting nature of work, care of healthy people* and *professionalism*. These categories embodied factors that were viewed as essential in being the best midwife and have been identified as key components of being ‘with woman’ (Bradfield et al. 2019:6). The categories have also been identified as essential in transitioning into specialty practice with a sense of belonging to the team and organization (Harvey et al. 2019:10). The attributes that participants identified as contributing to pride in being a midwife have been identified as central to advanced midwifery practice (Goemaes et al. 2016:36).

The relationship with physicians and authorities was important sentiment in how midwives viewed the second theme, **regulations and independent function**. Midwives in public hospitals generally felt that they could function independently in the absence of physicians but that hospital authorities did not always allow them to make decisions or create change. Regardless of private or public employment setting, midwives voiced lack of appropriate utilisation. For those in the private sector, midwives were restricted from practicing consistent with preparation; midwives in the public facilities were hampered from practicing a midwifery model of care because of system burdens and poor resource appropriation.

The third and fourth themes were **resource availability** and **work burden**. A *lack of resources*, such as equipment and supplies, access to technology and especially the *shortage of physicians and midwives*, together with large *influx of patients* – many immigrants, had a serious effect on the practice and care provided by midwives. Prior research has found that recent immigration, younger age and little formal education increased the risk for negative birth experiences for women in public facilities (Oosthuizen et al. 2017:1). Lack of resources noticed in this study no doubt contributed to a central theme, which underscored the lack of time midwives spend with women. This ‘time poverty’ report has been confirmed by patients in other research, demonstrating limited patient participation and collaboration with midwives (Boyle, Thomas & Brooks 2016:26). The *midwife-to-patient ratio* was a significant factor in labour wards where midwives literally ran from one birth to the next, a finding corroborated by mothers’ reports of childbirth experiences in South Africa (Hastings-Tolsma et al. 2018:5). Finally, there was widespread belief that *administrative support* for midwives was often lacking, as was space for providing *family-centred care.* Both administrative support and space for family engagement during labour were identified as crucial for midwifery functioning and demonstrated adequate resource availability. These components have also been identified as important to women crucial to the maternity health needs of women (Mohale, Sweet & Graham 2017:304).

The fifth theme centred on the **image of the midwife** influenced by *respectful care, patient trust* and *public perception* of the midwife. Current mainstream maternity services focus on medical risk status rather than on the individual woman – in total contradiction to a midwifery model of care and women know exactly what they want from their caregivers (Davison et al. 2015:774–775). Furthermore, mothers have also reported that attending midwives had inadequate listening skills (Maputle & Nolte 2008:60). As women surrender to the birth process, midwives must be available through supportive actions and openness (Lundgren & Berg 2007:225) and serve as both anchor and companion (Lundgren 2004:368). Where there is shared decision-making, women express greater satisfaction with birth (VandeVusse 1999:49).

In interview of women giving birth in a public hospital in South Africa, narratives of distress were based on negative interpersonal relations with midwives, lack of information, neglect and abandonment and the absence of a labour companion (Chadwick et al. 2014:862). In the absence of midwifery attention, labouring women experience a sense of isolation and loneliness (Kruger & Schoombee 2010:90). Midwives in our research reported that support persons were not allowed with women in most public hospitals because of a lack of space and privacy concerns for other patients. This means that if midwives were not present, most women were totally without support during labour and birth. Physical presence must be provided – particularly at transitional phases, if midwives are to engage with labouring women (Borrelli, Spiby & Walsh 2016:108). Where there is a sense of midwifery presence, physical and ontological safety and respectful care, women report a positive birth narrative (Chadwick 2019:6).

The importance of continuous presence of the midwife with women during labour has been emphasised in the literature. Whilst the overall influence of continuity of the carer on partnership with patient is unclear (Freeman 2006:39), continuous presence of the midwife has been associated with an increase in the woman’s sense of control and coping (Bohren et al. 2017:3), affecting a woman’s choice of pain relief (Aziato, Ohemeng & Omenyo 2016:1; Bohren et al. 2017:21; Howarth, Swain & Treharne 2011:92) and is important for maternal-child attachment and well-being of the new family (Aune, Amundsen & Aas 2014:93; Howarth et al. 2011:93). It is proposed that midwives who work ‘with’ woman in labour, experience a greater sense of job satisfaction and find their work emotionally rewarding (Hunter 2004:270–271). Furthermore, midwives regard continuous presence and support as essential in providing quality care during labour (Aune et al. 2014:93).

The participants in this research were aware that midwives in the public setting were often accused of disrespectful, non-caring behaviour towards women in labour. The fact that these same complaints have not been observed in the private or independent maternity hospital settings suggests that the difference in empathy is likely related to the climate of the institution (Miller & McLoughlin 2014:813). Midwives interviewed by Kruger and Schoombee (2010:97) spoke of becoming so angry and frustrated that they had violent feelings towards patients. It was mostly the ‘disobedient’ or ‘resistant’ patients that they felt had to be treated harshly. Burdens in the public system are likely a root cause of midwifery behaviour. It has been observed that the practice of midwives is moderated – even distorted, by context. Working in systems that are dominated by a medical model create the use of interventions antithetical to the philosophy of midwifery care; such philosophical dissonance may contribute to high exodus rates (Downe, Simpson & Trafford 2007:137–138).

Mistreatment of women in labour – whatever the reason – is a global health problem in need of directed intervention (Bohren et al. 2015:23; Vogel et al. 2016:671). A potentially important starting point would be implementation of tools to measure women’s perception of respectful maternity care (Sheferaw, Mengesha & Wase 2016:7–8). Use of such tools would heighten awareness of patient satisfaction with care during birth, as well as provide data for midwifery use to create needed change.

The trusting relationship between the midwife and the woman during childbirth has received emphasis in the literature. Interpersonal competence is one of the five main aspects in Halldorsdottir and Karlsdottir’s (2011:810–811) evolving theory on the empowerment of childbearing women. Many authors have emphasised that the quality of the relationship/partnership is a vital factor in the quality of midwifery care and key to a positive labour experience (Bo’Borrelli 2014:7–8; Hunter 2004:269–270; Lundgren & Berg 2007:226). In our research, midwives reported being unable to form a relationship with labouring women in both the public and private setting. A partnership case loading model should be considered as a strategy to foster a relationship between midwives and patients and promote patient decision-making (Boyle et al. 2016:27). In addition, models with the potential to reduce in-patient volume should be explored as a means of increasing midwifery availability and social support for women. One model that has been successful in sub-Saharan Africa is the maternity waiting home (Kaiser et al. 2019:9), although further research is needed to determine its impact on midwives working in overburdened and under-resourced systems and where there is failure to use midwives consistent with education.

The long working hours, too many patients, a midwife shortage and the lack of resources, are all factors that affect working conditions (Manyisa & Van Aswegen 2017:36) and lead to exhaustion amongst midwives. These factors likely create high levels of persistent stress that contribute to midwifery attitudes, professional presentation and interactions with patients. Chronic stress levels can adversely impact midwifery health and have been linked with depression, hypertension, diabetes and obesity (Schultz, Chao & McGinnis 2009:2). This study did not inquire about personal health status of the midwife; research is needed in this area.

The fact that midwives leave patients in labour alone for long periods of time, prevents the provision of quality care. Midwives with high numbers of patients cannot assess patients regularly, which may contribute to the high numbers of maternal mortality and morbidity, as well as medico-legal cases. The inability to be *with woman* during labour and birth may well be an important component in failure to rescue from both unnecessary intervention (Hastings-Tolsma & Nolte 2014:587) promoting respectful normal birth.

Midwives the world over consider their relationship with childbearing woman as a major source of job motivation and satisfaction (Bloxosome et al. 2019; Kirkham et al. 2006). The basis of midwifery education, the *midwifery model of care*, is in direct contradiction to findings in this research. This model emphasises continuity of care by a midwife who is known and trusted and who employs watchful waiting (Hatem et al. 2008). The midwifery model of care supports the normalcy of pregnancy and birth and focusses on the natural, physiologic process of birth through vigilance (clinical skills and judgement, knowledge of self and limits, clinical objectivity, decisiveness, confidence, intelligence and intellectual curiosity), attention to detail (empowering women, integrity and honesty, humility, realistic, gentle, warmth, nurturing, understanding and supportive) and respecting the uniqueness of the woman (family-centred care, tolerance non-judgmental, compassion, interest in others, flexibility) (Bo’Borrelli 2014:8–10; Kennedy 2000:8). A midwifery model of care reduces the likelihood of intervention and makes it more likely that women are satisfied with their care (Sandall et al. 2016); neither public nor private settings in this study afforded midwives the opportunity to provide such care and is essential for adequate support of women and the promotion of physiologic birth (Stark, Remynse & Zwelling 2016).

Providing women-centred care has been found to be an important construct for midwives to be *with woman* (Bradfield et al. 2019). It is important that factors that threaten the nurse–midwife relationship and diminish the midwife’s role in being ‘with woman’ be addressed. This knowledge is urgently needed as it relates to the clinical education of midwives, which largely occurs in public facilities in South Africa. Care in those facilities may fail to promote relationship-mediated being and such care may best be cultivated in other settings (Borrelli et al. 2016:108). Where intentionally and authentically present, midwives are empowered by responses from women, promoting growth and self-confidence (Thelin, Lundgre & Hermansson 2014). Furthermore, student exposure to how midwives make clinical decisions significantly influences future professional behaviour (Daemers et al. 2017:11). An additional important strategy to consider is increasing student exposure to out-of-hospital birth that may better promote care practices supportive of labour and physiologic birth (Zinsser, Stoll & Gross 2016:98). Finally, research is needed to reveal how women view physiologic birth, how midwives support that desire (Darra & Murphy 2016) and ensure the shared decision-making desired by labouring women (Downe et al. 2018). These shifts, along with the development of guidelines detailing respectful midwifery care (Vogel et al. 2016), could promote WHO recommendations for health promotion during birth (WHO 2015b).

### Need for institutional change

Two types of midwifery practice have been identified in the literature: rites of passage where women’s needs during labour and birth are managed and rites of protection where well-being and labour progress are assessed, disrupting aloneness and reinforcing external wisdom (Reed, Rowe & Barnes 2016:276). There is clear need for institutional change to support such midwifery practice, irrespective of the practice setting. For midwives in private settings, scope of practice was largely limited to care provided by obstetric nurses. When practicing in the public setting, midwives were faced with excessive organisational demands and reduced bed capacity, which promoted disconnection of midwives from labouring women (Shallow, Deery & Kirkham 2018:69). Only when working in the independent maternity hospital setting was there observed to be women-centred care with a strong interpersonal context consistent with the literature (Fontein-Kuipers, De Groot & Van Staa 2018). Midwifery care in the later setting should serve as the gold standard for structuring midwifery services in the institutional setting. Restructuring midwifery practice and development of workforce policy that promotes midwifery job satisfaction has the potential to promote retention of midwives at a time of significant shortage (Bloxsome, Bayes & Ireson 2020:386). Where there is an improved caring environment with a user-friendly infrastructure that provides reverence to adequate space, privacy, provision of supplies and staffing of midwives, care of women can be improved (Borrelli et al. 2016:103).

Reference to the *green epaulet girls* of years past is an apt metaphor for the changes needed in midwifery. Participants in this research viewed midwifery preparation as less rigorous and with less emphasis on woman-centred care than in the past, which is consistent with the literature (Webbelink 2019:150–151). Furthermore, participants from all settings were acutely aware of factors that restricted enactment of the full scope of midwifery care. Concerted effort to reclaim the midwifery model of care has the potential to redirect midwifery services and to improve the image of midwives held by patients, other professionals and the public.

### Need for health policy change

It is unclear how many midwives currently work in South Africa because those completing basic programmes are licensed as both a nurse and a midwife. There is an urgent need for an accurate and comprehensive midwifery workforce study verifying the number and availability, consistent with the WHO mandate for nations to clarify midwifery roles and scopes of practice. The WHO has mandated implementation of data collection and information systems to enable reliable reporting on the midwifery workforce status (WHO 2016). Comprehensive data collection is essential for determining availability, accessibility, acceptability and quality of the midwifery workforce (UNFPA 2014:30) and for workforce planning (Pozo-Martin et al. 2017:12). There is also a demonstrable need for redirection of health policies that engage midwives as key stakeholders, particularly related to the mistreatment of women (Jewkes & Penn-Kekana 2015:2–3) with midwives an integral part of the dialogue to change the dynamic of birth (Bradley et al. 2016:157). Maternity systems that limit the capacity of midwives to enact their wisdom, skilled practice and enacted vocation likely provide suboptimal care (Downe et al. 2007:133–136). Policies that reinforce midwives as low status workers in the health system and a failure to recognise them as professionals, effectively constitute disrespect and abuse of midwives. These factors are important to examine if the maternity care environment is to be improved (Bradley et al. 2019:1). Such change, along with an upscaling of midwifery education and a reallocation of resources and higher salaries, can enable delivery of care consistent with the midwifery model (Lori, Stalls & Rominsk 2015:6) and promote improved perinatal outcomes. Furthermore, midwives need to be engaged to the full extent of their preparation in private facilities; functioning essentially as obstetric nurses is inconsistent with educational preparation and denies women access to care promoting physiologic birth and shared decision-making. And finally, there should be reconsideration of policies, which mandate facility-based birth where psycho-emotional support from midwives is absent (Bradley et al. 2016:157). Midwifery models that reconsider birth setting whilst still promoting safe and satisfying birthing are urgently needed.

**FIGURE 1:**
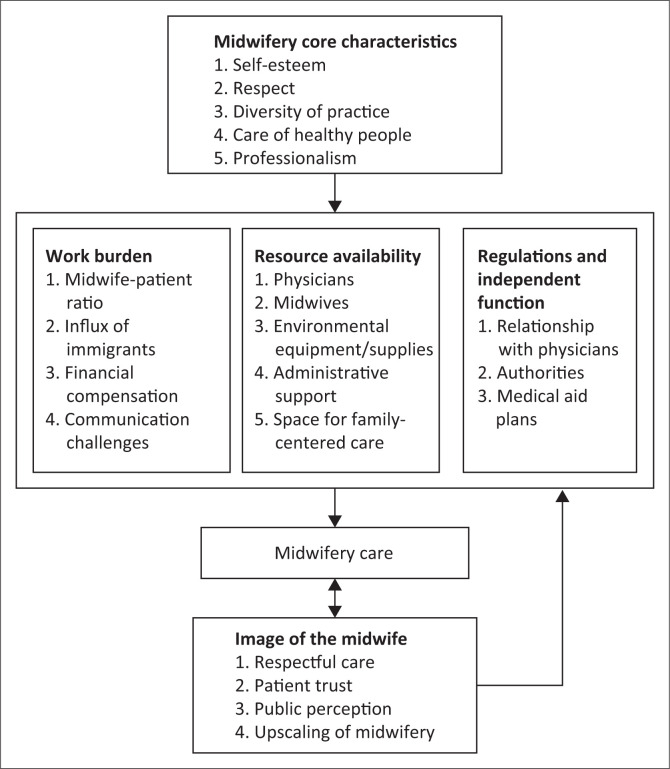
Overview: The general structure for reclaiming midwifery.

In summary, participants generally expressed both personal and altruistic reasons for working as a midwife. Where there were increased regulations, less independence and poorer compensation, greater role discontent emerged. Access to resources impacted execution of care consistent with personal values. Inadequate resources coupled with overburdened systems, contributed to midwifery care that was viewed by patients and the public as mistrustful and disrespectful. The interrelationship of key themes and categories important in reclaiming midwifery care in a manner that embraces the values demonstrated by the ‘green epaulet girls’ is depicted (Figure 1). It is interesting to note that the themes identified in this research were essentially the same as those noted in research conducted in South Africa over two decades ago (Jewkes et al. 1998:1781), underscoring the need for focussed attention in correcting underlying problems.

### Limitations

The study sample size was small and utilised convenience sampling. Despite saturation being reached, it is possible that additional interviews of midwives in each of the settings may have yielded additional information. Member checking was not performed and the researchers’ interpretation of interview data may not have accurately reflected participant feeling or meaning. Specifically, this research included only one midwife working in an independent maternity hospital setting and is a notable limitation in the application of findings.

## Conclusions

This research provides insight into the experiences of midwives in providing care to labouring women in varied healthcare settings. Challenges influential in providing care consistent with the midwifery model were described and were influential in the ability of midwives to address the health needs of women. The resulting stress that was experienced likely contributes to midwives leaving the profession (Banovcinova & Baskova 2014:253). Both in South Africa and globally, the need for midwifery care has never been greater and attention to midwifery services is urgently needed. In particular, the integration of educated, regulated and licensed midwives with a professional passion is needed – all factors in improved quality of care and sustained decreases in maternal and newborn mortality (Filby, McConville & Portela 2016:14).

Shortcomings in midwifery care may best be addressed through an upscaling of education, attention to quality of care beyond focus on mortality, emphasis on the setting for care (Homer et al. 2014:1153) and a reduction in stress-related factors (Leinweber & Rowe 2010:85). Midwives have been challenged to protect and promote women from health systems where women are ill-served (Hastings-Tolsma & Nolte 2014:592). The leadership and culture of health systems carry clear burden for the current crisis in caring and are notably blameworthy (Scott 2014).

Maternity care systems require cultural reform at a structural level to enable an opportunity for midwives to both reframe their relationship with patients and work more effectively with inter-professional teams (Dove & Muir-Cochrane 2014:1070). Promotion of midwifery processes offers a crucial vehicle for improving maternal-infant outcomes with midwives having the ability to enhance, humanise and empower women during the critical time of childbirth (Halldorsdottir & Karlsdottir 2011). This vulnerable time mandates a compassionate, caring environment with targeted processes that empower midwives as key stakeholders responsible for protecting the sacred space of birth (Fahy, Foureur & Hastie 2008). Such change will promote a reclaiming of the historical midwifery image of those *green epaulet girls* … those midwives of earlier times who embodied the respect, recognition and reward so richly deserved. It is an image sorely needed by the women midwives serve.
